# Relationship between Muscle Mass, Bone Density and Vascular Calcifications in Elderly People with SARS-CoV-2 Pneumonia

**DOI:** 10.3390/jcm12062372

**Published:** 2023-03-19

**Authors:** Rossella Del Toro, Francesco Palmese, Francesco Feletti, Gianluca Zani, Maria Teresa Minguzzi, Ernesto Maddaloni, Nicola Napoli, Giorgio Bedogni, Marco Domenicali

**Affiliations:** 1 Department of Primary Health Care, Internal Medicine Unit Addressed to Frailty and Aging, Santa Maria delle Croci Hospital, AUSL Romagna, 48121 Ravenna, Italy; rossella.deltoro@auslromagna.it (R.D.T.); giorgio.bedogni@unibo.it (G.B.); m.domenicali@unibo.it (M.D.); 2 Department of Medical and Surgical Sciences, Alma Mater Studiorum University of Bologna, 40126 Bologna, Italy; 3 Department of Translational Medicine and for Romagna, University of Ferrara, 44121 Ferrara, Italy; 4 Department of Diagnostic Imaging, Radiology Unit, Santa Maria delle Croci Hospital, AUSL Romagna, 48121 Ravenna, Italy; 5 Department of Anesthesia and Intensive Care, Santa Maria delle Croci Hospital, AUSL Romagna, 48121 Ravenna, Italy; 6 Department of Experimental Medicine, Sapienza University of Rome, 00185 Rome, Italy; 7 Department of Medicine and Surgery, Research Unit of Endocrinology and Diabetes, Campus Bio-Medico University of Rome, 00128 Rome, Italy

**Keywords:** frailty, SARS-CoV-2, muscle mass, bone density, vascular calcifications, mortality, computed tomography, diagnostic imaging

## Abstract

Background: Little is known about the changes in organs and tissues that may make elder patients more vulnerable to acute stressors such as SARS-CoV-2 infection. Methods: In 80 consecutive elderly patients with SARS-CoV-2 infection, we evaluated the association between the descending thoracic aorta calcium score, L1 bone density and T12 skeletal muscle density measured on the same scan by high-resolution computed tomography. Results: At median regression, the ln-transformed DTA calcium score was inversely associated with L1 bone density (−0.02, 95%CI −0.04 to −0.01 ln-Agatston units for an increase of 1 HU) and with T12 muscle density (−0.03, −0.06 to −0.001 ln-Agatston units for an increase of 1 HU). At penalized logistic regression, an increase of 1 ln-Agatston unit of DTA calcium score was associated with an OR of death of 1.480 (1.022 to 2.145), one of 1 HU of bone density with an OR of 0.981 (0.966 to 0.996) and one of 1 HU of muscle density with an OR of 0.973 (0.948 to 0.999). These relationships disappeared after correction for age and age was the stronger predictor of body composition and death. Conclusions: Age has a big effect on the relationship between vascular calcifications, L1 bone density and T12 muscle density and on their relationship with the odds of dying.

## 1. Introduction

Frailty is a condition characterized by a decline in several homeostatic systems, which makes a person more vulnerable to stressors and puts them at risk for health problems [1,2]. Evidence-practice gaps in the clinical care of frailty have been addressed by several strategies. Some studies explore links between biological changes and changes in organs and tissues that cause frailty, and others aim at identifying frailty biomarkers [3].

The SARS-CoV-2 pandemic has opened a new chapter, with several studies indicating that SARS-CoV-2 mortality is linked to frailty in patients aged ≥ 65 [4,5,6,7,8,9]. Nowadays, vaccines have made the clinical picture of SARS-CoV-2 infection much better [10]. However, much of the world population is still unvaccinated, and future variants might cause severe forms of pneumonia [11]. We had never seen such a large-scale exposure of the elderly population of industrialized countries to an infectious agent capable of generating such high levels of inflammation. Understanding what happened in the fragile population may allow for new strategies beyond the treatment of SARS-CoV-2. A recent study that looked at 63 hospitals in 11 European countries and included 2434 patients found that the Clinical Frailty Scale is a good risk marker for hospital mortality in adults with dementia [12].

However, there remains a gap in the literature regarding pre-existing changes in organs and tissues, which may drive vulnerability and reduce resistance to stressors as acute as SARS-CoV-2. With this in mind, researchers should investigate body composition and sarcopenia as markers of frailty in the elderly and as a predictor of poor prognosis in SARS-CoV-2 pneumonia. Recent data show that low muscle mass and high visceral fat are predictors of negative outcomes in SARS-CoV-2 patients [13,14].

Arterial calcification is strongly associated with atherosclerosis and has been proposed as a biological marker of aging. Calcium efflux from bone increases with age-related bone loss, which reduces bone mineral density. Age-related increases in calcium efflux in the arterial wall progressively stiffen blood vessels, but the relations between these processes have to be further explored [15]. Thomas et al. found that the calcium content of the descending thoracic aorta (DTAC) was associated with non-cardiovascular disease (CVD) mortality, including chronic obstructive pulmonary disease, hip fracture and pneumonia [16]. Furthermore, cardiovascular calcification and osteoporosis follow the same pathogenic pathway, and animal models confirm the existence of abnormalities linked to aging. Mice with defects in klotho gene expression show a short lifespan, emphysema, osteoporosis, and the calcification of the medial layer of the aorta [17,18].

We hypothesized that frailty in the elderly involves pathways common to muscle mass, bone density and blood vessel calcification. On that basis, the aim of this study was to evaluate the relationship between paravertebral skeletal muscle mass, lumbar vertebral bone density and thoracic aortic calcifications measured on the same high-resolution computed tomography (HRCT) scan and their association with death in patients with SARS-CoV-2 pneumonia.

## 2. Materials and Methods

### 2.1. Study Design

This is a cross-sectional study of patients aged ≥ 65 years with SARS-CoV-2 who were admitted to the Department of Internal Medicine and the Intensive Care Unit of Santa Maria delle Croci Hospital (Ravenna, Italy) from 1 February to 31 March 2021 during the second wave of the SARS-CoV-2 pandemic. To be eligible for the study, the patients had to have SARS-CoV-2-related pneumonia [19], including swab tests positive for coronavirus and radiological evidence of interstitial pneumonia; furthermore, each patient had to have undergone an HRCT of the chest comprising all the regions of interest for the present study. Oncological disease and immunosuppressive therapy were reasons for exclusion from the study. The study was conducted in accordance with the Declaration of Helsinki and ethical approval was obtained from Comitato Etico della Romagna (CEROM protocol number 10263/2021 I.5/305 approved on 10 December 2021); informed consent was obtained from all subjects.

### 2.2. Laboratory Assessment

Blood was collected on the first day of hospitalization for routine biochemical analysis, including a complete blood count, high-sensitivity C-reactive protein, alanine aminotransferase (ALT), bilirubin, lactic dehydrogenase (LDH) and prothrombin time.

### 2.3. HRCT

HRCT was performed with a Philips Brilliant CT 64-slice system at a resolution of 0.977 × 0.977 mm. The images were taken from the PACS archive and transferred to the Philips Intellispace Portal 9.0 console and were preliminarily post-processed through the Multi-Modality Advanced Vessels Analysis protocol and specific MPR reconstructions.

#### 2.3.1. HRCT—Calcium Content of Descending Thoracic Aorta

**Figure 1:**
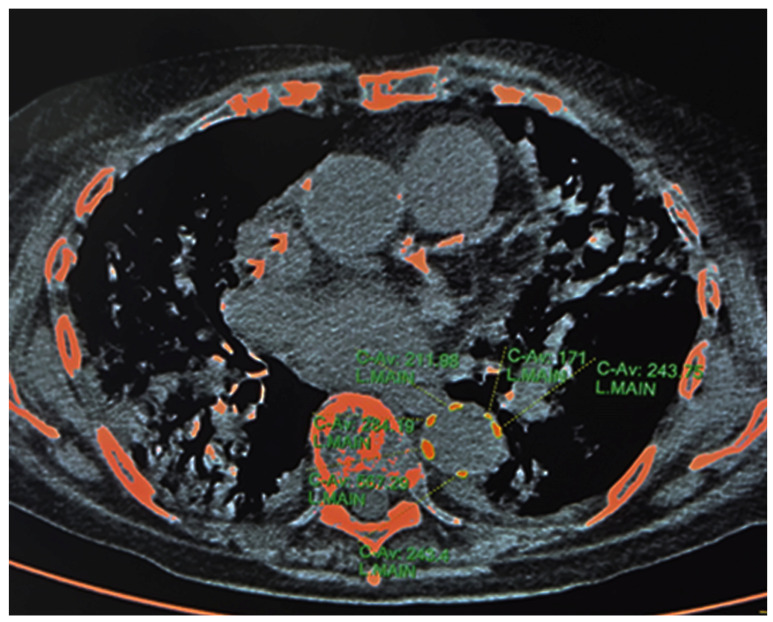
Calcifications of descending thoracic aorta—Agatston Score. Credits: AUSL Romagna.

DTAC was defined as the amount of calcium within the wall of the descending thoracic aorta as measured by HRCT [16,20]. The calcified atheromas of interest were selected manually. We included the aortic tract located between an upper plane passing through the bifurcation of the pulmonary artery and a lower one passing through the apex of the heart [16]. The system automatically calculated the Agatson score by using a threshold of 130 HU [21] (Figure 1).

#### 2.3.2. HRCT—T12 Paravertebral Muscle Area and Density

**Figure 2:**
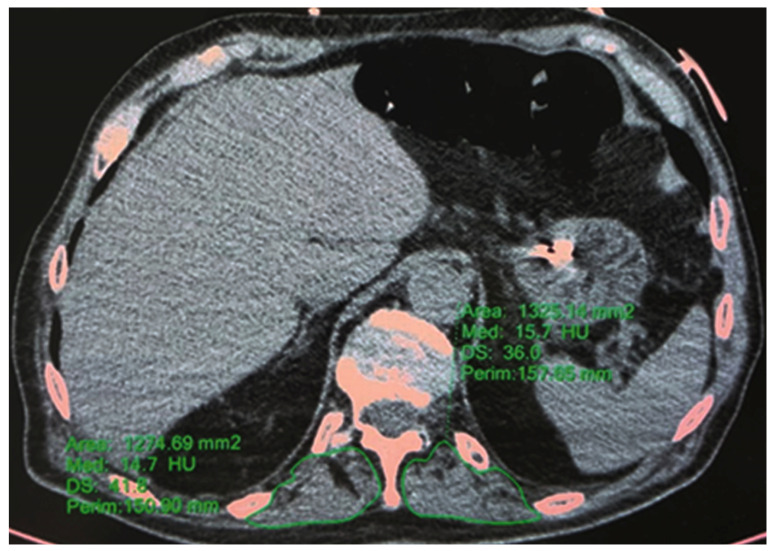
T12 paravertebral spinal muscle area and density. Credits: AUSL Romagna.

The T12 skeletal muscle area was defined as muscle tissue located posterior to the T12 spine and ribs and lateral to the lateral borders of the erector spinal muscles. The T12 muscle density was measured using Hounsfield units (HU) [14,22]. A single slice passing through the body of T12 was used to measure the paravertebral muscles. The T12 skeletal muscle mass was calculated as the sum of the right and left dorsal areas, whereas the T12 skeletal muscle density was calculated as the mean of the right and left dorsal radiodensities. The muscles’ perimeter was drawn on both sides using the multi-modality viewer’s “ellipse” function (Figure 2). Low skeletal muscle area is linked to poor prognosis in patients with various types of cancer. For instance, sarcopenia is an independent predictor of poor postoperative survival in patients with lung cancer [22]. An association has been reported between skeletal muscle density and mortality in mechanically ventilated patients [23]. Additionally, a low T12 skeletal muscle area has been reported as an independent predictor of in-hospital mortality and long-term survival among patients with community-acquired pneumonia [24].

#### 2.3.3. HRCT—L1 Bone Mineral Density

**Figure 3:**
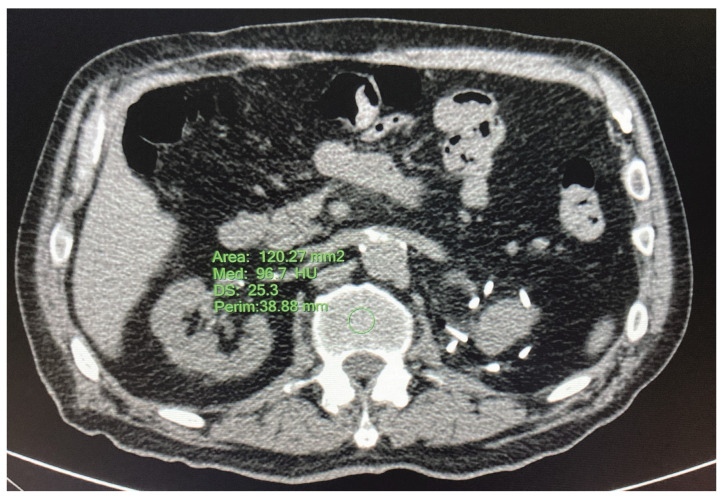
L1 bone mineral density. Credits: AUSL Romagna.

The L1 vertebra is usually included in chest and abdominal CT scans, is easily identified and is useful for retrospective studies [25]. The L1 vertebral bone mineral density was measured using CT attenuation in HU by drawing a circle with a diameter of 1 cm in the center of the vertebral body of L1 [26] (Figure 3).

### 2.4. Statistical Analysis

Most continuous variables were not Gaussian distributed, and all are reported as median (50th percentile) and interquartile intervals (IQI, 25th and 75th percentiles). Discrete variables are reported as the number and proportion of subjects with the characteristic of interest. The DTAC Agatston score was transformed for analysis by adding 0.01 to its value, ranging from 0 to 14,526, and log-transformed by taking the natural logarithm (ln) of the ensuing value. This reduced its skewness and allowed DTAC to meet the assumptions made by the median or logistic regression models where it was used as a response or predictor variable.

We used median regression with heteroscedasticity-robust standard errors to quantify the association between: (1) Ln DTAC score and bone density (continuous predictor); (2) Ln DTAC score and muscle density (continuous predictor); (3) muscle density and bone density (continuous predictor); and (4) muscle area and bone density (continuous predictor) [27,28]. We pre-specified three median regression models for each predictor as follows: M1 containing only the continuous predictor of interest; M2 adding age (continuous, years/10) as a predictor to M1; and M3 adding gender (discrete, 0 = female; 1 = male) as predictors to M2 [29]. Such models were pre-specified because of the known effects of age and gender on DTAC score, bone density and muscle density. Univariable and multivariable fractional polynomials were used to evaluate whether the relationship of the response variable with the continuous predictors was linear, which was found to be so in all models [29].

We used penalized logistic regression to evaluate the association between the occurrence of death during the hospital stay and the predictors of interest (DTAC score, muscle density and bone density). Penalized logistic regression was used because of the low absolute number of deaths [30,31]. We pre-specified three logistic regression models for each predictor as follows: M1 containing only the predictor (continuous) of interest; M2 adding age (continuous, years/10) as the predictor to M1; and M3 adding gender (discrete, 0 = female; 1 = male) as predictors to M2. Such models were pre-specified because of the known effect of age and gender on the risk of death. Univariable and multivariable fractional polynomials were used to evaluate whether the relationship of the logit of death with continuous predictors was linear, which was found to be so in all models [29]. Statistical analysis was performed using Stata 17.0 (Stata Corporation, College Station, TX, USA).

## 3. Results

### 3.1. Baseline Features of the Patients

Table 1 gives the baseline features of the patients.

The patients had normal median values of blood count, electrolytes, renal function, prothrombin time and lactate dehydrogenase. Nevertheless, the frequent presence of lymphopenia is an indicator of the severity of the underlying SARS-CoV-2 infection [32].

### 3.2. Association between DTAC Score, Bone Density, Muscle Density and Muscle Area

Table 2 gives the univariable and multivariable median regression models used to evaluate the associations of interest.

At median regression, an increase of 1 HU of bone density was associated with a decrease of 0.02 ln-Agatston units of DTAC score (Model M1a), but this relationship disappeared after age (Model M1b) and age and sex (Model 1c) were taken into account. An increase of 1 HU of muscle density was associated with a decrease of 0.03 ln-Agatston units of DTA calcium score (Model M2a) but this relationship disappeared after age (Model M2b) and age and sex (Model 2c) were taken into account. An increase of 1 HU of bone density was associated with an increase of 0.13 HU of muscle density (Model 3a) but this relationship disappeared after age (Model M3b) and age and sex (Model M3c) were taken into account. There was no association between muscle area and bone density (Models 4a–4c). Importantly, age was the strongest predictor of all outcomes. Sex added only to the prediction of muscle density from bone density.

### 3.3. Association between Death and DTAC Score, Bone Density, Muscle Density and Muscle Area

Sixteen out of 80 patients (20%) died during the hospital stay. Table 3 gives the univariable and multivariable penalized logistic regression models used to evaluate the association between death and ln DTA calcium score, bone density, and muscle density.

An increase of 1 ln-Agatston unit of DTA calcium score was associated with an OR of death of 1.480 (95%CI 1.022 to 2.145, Model M1a); however, the addition of age (Model M1b) and age and sex (Model M1c) to the model caused this association to disappear. An increase of 1 HU of bone density was associated with an OR of death of 0.981 (95%CI 0.966 to 0.996, Model 2a); however, the addition of age (Model M2b) and age and sex (Model M2c) to the model caused this association to disappear. An increase of 1 HU of muscle density was associated with an OR of death of 0.973 (95%CI 0.948 to 0.999, Model M3a); however, the addition of age (Model M3b) and age and sex (Model M3c) to the model caused this association to disappear. Importantly, age was the strongest predictor of death in all models and sex did not add to it.

## 4. Discussion

This study analyzed for the first time three different tissues on the same HRCT scan in patients with SARS-CoV-2 pneumonia. We found inverse relationships between DTAC score and bone density and DTAC score and muscle density. This relationship disappeared, however, after the contribution of age was taken into account. Importantly, the direct association between DTAC and death and the inverse associations between muscle density, bone density and death also disappeared after correction for age.

Even if this study is the first to employ the same HRCT scan to obtain measures of vascular calcifications, bone density and muscle density, it is not without limitations. The main limitation is that it was performed on a relatively low sample of subjects (N = 80) and, even if the death rate (20%, N = 16) was in keeping with the expectations, the absolute number of events was too low to obtain precise estimates of the effects sizes. However, we found clear evidence of a big effect of age on the relationship between vascular calcifications, bone density and muscle density, as well as their association with death. Moreover, the small sample size prevented us from performing a mediation analysis aimed at establishing the effect of age on the association between body composition measurements and between them and death.

There is increasing evidence that there is a link between vascular calcification and bone metabolism; coincidentally, aging is characterized by the development of osteoporosis and vascular disease. The seemingly contradictory association between bone demineralization and vascular mineralization is commonly referred to as the bone–vascular axis [33]. Several studies have demonstrated an inverse association between bone mineral density and vascular calcification. Furthermore, high bone turnover is associated with increased CV mortality in elderly individuals, regardless of age, gender, PTH serum levels or previous hip fractures [34]. The results of the present study show that the density of L1 trabecular bone is inversely associated with the calcium content of the descending thoracic aorta in elderly patients, but this association disappears after correction for age.

Giannini et al. reported that coronary, aortic and thoracic aortic calcium can be used to assess the risk of death in SARS-CoV-2 patients using non-gated CT [35]. In their study, total thoracic calcium, which included coronary, aortic valve and thoracic aortic calcium, was a stronger predictor of mortality than coronary artery calcium. Instead, we focused our investigation on assessing DTAC for two reasons. First, CT can help identify individuals with increased vulnerability to non-CVD-related morbidity and mortality, especially chronic obstructive pulmonary disease, hip fracture and pneumonia. Second, there is a prognostic difference between medial and intimal calcifications. Both atherosclerotic and non-atherosclerotic processes were demonstrated in the thoracic aorta by Abramowitz et al. [36]. Medial non-atherosclerotic calcification may reflect biological aging and is more frequently reported in the aorta than in the coronary arteries. This may explain the different associations of thoracic aortic and coronary artery calcifications with non-CVD mortality. By selecting only the descending segment of the thoracic aorta, we attempted to reduce the impact of known risk factors on CVD.

Older people often have bone loss which suggests the presence of osteoporosis, which is defined as a systemic skeletal disease with an increase in bone fragility and susceptibility to fractures [37]. The interaction between bone and muscle has been the focus of scientists’ attention for decades, not only because of its importance for the musculoskeletal system but also because of the complex chemical and metabolic interactions [38]. Genetic, endocrine and environmental factors are recognized to be the basis of sarcopenia and osteoporosis, both of which are associated with aging. The loss of bone mineral density appears to be coincidental with decreased muscle mass, strength and function, and it is accepted as a single disease called osteosarcopenia. An increased risk of falls, fractures, frailty, and mortality is associated with osteosarcopenia. Our results are consistent with the literature, which supports a direct association between bone density and muscle mass. We found, however, that the relationship disappeared after correction for age.

Recent studies have shown that muscle density is related to muscle strength, and its measurement could be important for diagnosing and screening for sarcopenia [39]. In a subset of the obese population, osteopenia and osteoporosis can be found simultaneously, giving rise to osteosarcopenic obesity, which has worse health outcomes [40]. Both sarcopenia and obesity have been identified as risk factors for mortality in SARS-CoV-2 infection. In agreement with the hypothesized synchronous trend of bone loss and sarcopenia, we found an inverse correlation between DTAC score and T12 muscle density in the present study. After correcting for age, however, the correlation was lost.

## 5. Conclusions

In conclusion, we examined the association between descending aorta calcifications, L1 bone mineral density and T12 muscle density on the same HRCT scan in elderly people with SARS-CoV-2 pneumonia. Vascular calcifications were inversely related to bone mineral density and muscle density, and bone and muscle density were directly related, but none of these findings stayed after adjusting for age. Importantly, the odds of death were directly associated with DTAC and inversely associated with L1 bone mineral density and T12 muscle density, but this association was lost after correction for age. Therefore, age is an important factor that should be taken into consideration by further studies in this area.

## Figures and Tables

**Table 1:** Baseline features of the patients.

Male Sex	41 (51%)
Age (years)	79 (73; 85)
Age (years/10)	
6	11 (14%)
7	34 (42%)
8	29 (36%)
9	6 (8%)
White blood cells (10^9^/L)	6.69 (4.96; 9.65)
Lymphocytes (10^9^/L)	0.76 (0.55; 1.13)
Neutrophils (10^9^/L)	5.64 (3.80; 8.32)
Platelets (10^9^/L)	210 (156; 279)
Hemoglobin (g/dL)	12 (11; 13)
Prothrombin time (INR) *	1.1 (1.0; 1.2)
ALT (U/L) **	21 (14; 34)
Total bilirubin (mg/dL) *	0.4 (0.3; 0.6)
LDH (U/L) **	325 (274; 401)
C-reactive protein (mg/dL)	79 (40; 123)
DTA calcium score (Agatston units)	984 (51; 2991)
Ln DTA calcium score (Agatston units)	7 (4; 8)
Muscle density T12 (HU)	20 (4; 29)
Muscle area (cm^2^)	2820 (2234; 3191)
Bone density L1 (HU)	86 (63; 117)

Measurements of the 80 patients. Values are numbers (percentages) for discrete variables and median (interquartile interval) for continuous variables. Abbreviations: Ln = natural logarithm. * N = 79; ** N = 78.

**Table 2:** Univariable and multivariable median regression models used to evaluate the association between ln DTA calcium score and bone density, ln DTA calcium score and muscle density, muscle density and bone density and muscle area and bone density.

	ln DTA calcium score		
	M1a	M1b	M1c
Bone density L1 (HU)	−0.02 ** [−0.04 to −0.01]	−0.00 [−0.02 to 0.02]	0.00 [−0.02 to 0.03]
Age (years)/10		2.12 *** [0.90 to 3.33]	2.28 ** [0.81 to 3.74]
Male sex			−0.56 [−2.35 to 1.23]
Muscle density T12 (HU)			
Intercept	8.71 *** [7.54 to 9.88]	−10.64 [−21.84 to 0.56]	−12.25 [−25.55 to 1.04]
	**ln DTA calcium score**		
	M2a	M2b	M2c
Age (years)/10		1.89 ** [0.78 to 3.01]	1.91 ** [0.72 to 3.11]
Male sex			−0.40 [−1.93 to 1.13]
Muscle density T12 (HU)	−0.03 * [−0.06 to −0.001]	−0.01 [−0.05 to 0.03]	−0.01 [−0.04 to 0.03]
Intercept	7.15 *** [6.45 to 7.85]	−9.01 [−18.50 to 0.48]	−8.92 [−18.99 to 1.15]
	**T12 Muscle density**		
	M3a	M3b	M3c
Bone density L1 (HU)	0.13 * [0.02 to 0.24]	0.08 [−0.11 to 0.26]	−0.03 [−0.15 to 0.10]
Age (years)/10		−5.74 [−15.77 to 4.30]	−10.89 ** [−18.87 to −2.90]
Male sex			11.05 * [2.25 to 19.86]
Intercept	7.93 [−3.12 to 18.98]	56.77 [−33.29 to 146.83]	98.58 ** [29.27 to 167.89]
	**T12 Muscle area**		
	M4a	M4b	M4c
Bone density L1 (HU)	1.37 [−3.91 to 6.65]	1.23 [−4.45 to 6.90]	−3.49 [−9.08 to 2.10]
Age (years)/10		−133.60 [−429.37 to 162.17]	−255.17 [−514.86 to 4.52]
Male sex			696.00 ** [276.35 to 1115.65]
Intercept	2715.30 *** [2198.35 to 3232.26]	3700.20 ** [1184.68 to 6215.72]	4745.28 *** [2526.68 to 6963.89]

Values are regression coefficients and 95% confidence intervals from median regression with heteroskedasticity-robust standard errors; * *p* < 0.05, ** *p* < 0.01, *** *p* < 0.001.

**Table 3:** Univariable and multivariable penalized logistic regression models used to evaluate the association between death and ln DTA calcium score, bone density, and muscle density.

	M1a	M1b	M1c
Ln DTA calcium score (Agatston units)	1.480 * [1.022,2.145]	1.225 [0.833,1.802]	1.206 [0.832,1.750]
Age (years)/10		3.177 * [1.201,8.406]	3.066 * [1.178,7.978]
Male sex			0.747 [0.227,2.460]
	**M2a**	**M2b**	**M2c**
Age (years)/10		3.530 ** [1.361,9.154]	3.464 ** [1.362,8.811]
Male sex			0.849 [0.249,2.898]
Bone density L1 (HU)	0.981* [0.966,0.996]	0.990 [0.973,1.008]	0.991 [0.974,1.009]
	**M2a**	**M2b**	**M2c**
Age (years)/10		4.023 ** [1.566,10.335]	3.926 ** [1.561,9.873]
Male sex			0.755 [0.228,2.492]
Muscle density T12 (HU)	0.973 * [0.948,0.999]	0.992 [0.965,1.021]	0.994 [0.966,1.022]

Values are odds ratios and 95% confidence intervals from penalized logistic regression; * *p* < 0.05, ** *p* < 0.01.

## Data Availability

The data are available on reasonable request.
